# Isolation and Identification of a Murine Norovirus Persistent Infection Strain in China

**DOI:** 10.3389/fvets.2020.571730

**Published:** 2020-12-01

**Authors:** Zhao Na, Jiang Bo, Yang Yifei, Cao Fuyuan, He Bin, Zhang Yanshu, Jin Huan, Su Jingliang, Li Shuang

**Affiliations:** ^1^The Experiment Animal Center, North China University of Science and Technology, Tangshan, China; ^2^Institute of Animal Husbandry and Veterinary Medicine, Beijing Academy of Agricultural and Forestry Sciences, Beijing, China; ^3^Institute of Chinese Materia Medica, China Academy of Chinese Medical Sciences, Beijing, China; ^4^Key Laboratory of Animal Epidemiology and Zoonosis, Ministry of Agriculture, College of Veterinary Medicine, China Agricultural University, Beijing, China

**Keywords:** murine norovirus, virus isolate, persistent infection, laboratory animals, C57 (C57BL/6J)

## Abstract

Murine Norovirus (MNV) is one of the most known viruses among viruses in mice. Because of the high prevalence of MNV in frequently used laboratory animals in biomedical researches, there is a significant impact of MNV. There may be different prevalence degrees and molecular characteristics of MNV in different regions around the world. Here, we reported an MNV strain “designated HBTS-1806” isolation from commercial mice's feces that caused a detectable cytopathic effect (CPE) in RAW264.7 cells. According to electron microscopy, the virus was 50–70 nm in diameter. The complete genome of HBTS-1806 is 7383 nucleotides with a structure similar to that of MNV reference strains. According to phylogenetic analysis on the basis of the whole genome, HBTS-1806 shared nucleotide sequence identities of 90.2–95.4% with other Chinese isolates reported. Analysis of amino acid sequence on the basis of ORF1 and ORF2 suggested that the isolated strain may be derived from recombination. Although no gross lesions or histopathological changes were found from mice infected with 5 × 10^5^ TCLD_50_ of MNV by oral gavage inoculation, the intestinal virus loads lasted 12 weeks, suggesting a persistent infection strain of MNV isolate in China.

## Introduction

As single-stranded and positive-sense RNA viruses, noroviruses belong to the family *Caliciviridae* and the genus *Norovirus*. Recently, noroviruses are divided into five genogroups (GI to GV) (1). The GI and GII viruses are the causes of most cases of acute non-bacterial gastroenteritis among all ages worldwide of human, causing great burden to public health (2, 3). Due to the lack of cell culture and small animal model systems research on the virus replication and pathogenesis of noroviruses have been difficult to carry out (4). Nevertheless, murine noroviruses (MNVs), categorized as GV, were discovered in conventionally housed mice at Washington University in a screen for novel human pathogens capable of infecting mice (5). This MNV can propagate in the RAW 264.7 cell line originated from mouse macrophages (4, 6). Therefore, MNV has been considered as an alternative model of human norovirus.

The MNV is non-enveloped, non-segmented virus and has ~7.5 kb pairs of bases that encode three open reading frames (ORFs): ORF1 encoding a large polyprotein cleaved to single non-structural proteins is encoded; ORF2 encoding the viral capsid protein (VP1) is encoded by ORF2; and ORF3 encoding a minor structural protein (VP2) (7–9). An additional ORF4 has also been found in the MNV genome (10). The original strain was called MNV-1, and then many MNV-1 strains have been isolated by different institutions around the world (11). The reports showed that all MNV strains isolated from laboratory mice around the world are of single genotype with ~13% nt and ~7% aa diversity in the ORF2 (VP1) region, indicating very limited genetic diversity when comparing with the human noroviruses (12).

The discovery of MNV-1 strain accelerated the development of assays for detection of antibody to MNV, so that serologic evidence is provided for a widespread distribution of this virus in laboratory mice (13, 14). Almost all mouse strains are liable to MNV infection (15, 16). In immunodeficient mice, MNV infection recapitulated various clinical symptoms such as hepatitis, focal interstitial pneumonia, and peritonitis (17–21). Propagateion could both wild-type and immunocompromised mice as an asymptomatic infection (22). Asymptomatically infected mice would cross-infect from institution to another and caused high prevalence of MNV among animal institutes (23–25). Undoubtedly, this infection is able to can cause detrimental effects to laboratory animals and bring biased experimental results. Therefore, the research on the epidemiology and etiology of MNV is of great particular importance. In this study, an MNV persistent infection strain from laboratory mice was identified and characterized in China.

## Materials and Methods

### Sample Collection

Three laboratory animal production companies in Beijing, Shandong, and Liaoning province respectively provided 160 C57BL/6J mice between March and September 2018. After mixing the feces of 10 individual mice from the same company, it was made into 10% suspensions in phosphate buffer (PBS, pH 7.2) was prepared and stored at −80°C.

### RT-PCR Detection of MNV

Detection of MNV in the samples of commercial laboratory mice were attempted with a MNV specific primer pair targeting a 396 bp region at the 5′ end of MNV ORF2 (VP1) (24). Briefly, 2 μl RNA as template was used in one step RT-PCR kit (Thermo Fisher Scientific) according to the manufacturer's protocol. Thermocycling conditions starts with reverse transcription at 42°C for 40 min (for the RT-PCR) followed by an initial incubation at 94°C for 2 min and 30 cycles of denaturation at 94°C for 40 s, annealing at 50°C or 40 s, and extension at 72°C for 10 min. PCR products were analyzed on 2% agarose gels in the presence of ethidium bromide.

### Virus Isolation and Identification

Adopting Eagle's medium (DMEM, Gibco, USA) modified by Dulbecco as growth medium (GM), supplemented with 10% heat-inactivated fetal calf serum, penicillin (250 units/ml, Gibco, USA) and streptomycin (250 mg/ml, Gibco, USA) were supplemented. Maintenance medium (MM) consists of DMEM supplemented with 2% heat-inactivated fetal calf serum, penicillin (250 units/ml, Gibco, USA) and streptomycin (250 mg/ml, Gibco, USA). The murine macrophage-like cell line RAW264.7 was supplied by the Cell Ceter of Basic Medical Sciences, Institute of Basic Medical Sciences, Chinese Academy of Medical Sciences (3111C0001CCC000146), which was used to perform virus isolation as described previously (26). Briefly, a 0.22-μm syringe filter (Millipore, USA) was used to further filter feces samples that are positive by reverse transcription-polymerase chain reaction (RT-PCR). A RAW264.7 cell monolayers was added with the feces samples in a 6-well tissue culture plate (Costar, Corning Inc., Corning, NY). After 60-min adsorption at the temperature of 37°C, MM was added to wash cells. Cultures were incubated at 37°C with 5%CO_2_, and its cytopathic effect (CPE) was checked every day. It was necessary to harvest and froze the cultures when CPE of the cells reaches 75%. A new RAW264.7 cell monolayer was infected by 20 ml of the resultant suspensio. According to the amount of associated CPE, the material was passed between 3 and 5 times. RT-PCR was used to test MNV in the cell culture supernatants.

### Electron Microscopy

Virus-infected RAW264.7 cells were fixed in 2.5% glutaraldehyde at 24 and 48 h after infection. Uranyl acetate and leadcitrate were used to stain the ultrathin sections. Then, a Hitachi TEM H-7500 was used to examine the samples as described previously (27).

### Animal Infection

Beijing Huafukang Bioscience Co., Ltd. (Beijing, China) which was certified MNV-free vendors provided female C57BL/6 mice. To investigate the efficiency of virus replication, the 6 week-old mice infected 5 × 10^5^ TCLD_50_ of MNV by oral gavage inoculation (*n* = 60), and the same volume of PBS (pH 7.2) was applied to treat the mice in the negative control group (*n* = 10). Food uptakes, body weight and clinical signs of infected mice were examined daily for 12 weeks. Ten infected mice were euthanized 2, 4, 6, 8, 10, and 12 weeks after infection. Heparinized blood and tissue samples from liver, spleen and appendix were collected from both infected and control mice. For each organ, one half was frozen at −80°C, and the other half was fixed in 10% neutral-buffered formalin. Paraffin-embedded tissue blocks were cut into 5 μm thick sections and stained with hematoxylin and eosin (HE) with standard methods.

### Virus Gene Amplification and Genome Sequencing

E.Z.N.A.®Viral RNA Kit (Omega, USA) was used to extract viral RNAs from MNV-infected RAW264.7 culture supernatant according to the manufacturer's instructions. The SuperScript III First-Strand Synthesis System for RT-PCR (Invitrogen, USA) was adopted to synthesize DNA. Different regions of the MNV genomes were amplified by applying 12 pairs of oligo nucleotide primers designed on basis of the sequence of MNV BJ 10-2062 strain (Table 1). After being purified and cloned into pMD18-T vector (TaKaRa, Japan), the PCR products were sequenced by Beijing Sunbiotech Co., Ltd. Lasergene software (DNAstar Inc., USA) was employed to assemble and analyze sequence data. Clustal X 2.1 was adopted to perform multiple sequence alignments. The MEGA 6 program was used to carry out phylogenetic analyses. The genome sequence of the isolated strain was registered in GenBank (the Accession Number MT358379).

**Table 1:** Oligonucleotide primers referred to in this study.

**Name**	**Sequence(5^**′**^-3^**′**^)**	Genomic postion^*^
1F	GTGAAATGAGGATGGCAACG	1-20
1282R	AGTTGGCACTCGTTCTTGAT	1263-1282
851F	AAACCTTCTGGCATCTGTGA	851-870
1985R	CAAGATGAAATTGATGTGGC	1966-1985
1790F	CATCATCATCACCACCAACC	1790-1809
3442R	ACCCAGGTGTTTCCTTTCTT	3423-3442
3229F	TTGTCGCTTCGGTCCTTGTT	3229-3248
4816 R	TGGTGATTGGGTCCTTTGGT	4797-4816
4388F	CCCTTCGCTGCTGGATGTTG	4368-4387
5967R	CACCTGACCCGTGCCTGATT	5948-5967
5454F	AGGGTCACTCACCACTGCTC	5454-5673
7382R	AAAATGCATCTAATTACTAC	7363-7382R

* *Location corresponds to position within the BJ 10-2062 (KM458057) genome*.

### Virus Quantitation by Real-Time PCR

The viral RNA detected by real-time PCR to monitor viral RNA in the organs and feces of mice. The primers used to detect MNV were MNV-S (5′-CCGCAGGAACGCTCAGCAG-3′) and R2 (5′-GGYTGAATGGGACGGCCTG-3′). The sequence of the TaqMan probe was 5′-FAM-ATGAGTGATGGCGCA-MGB-NFQ (28). A E.Z.N.A.®Total RNA Kit I (Omega, USA) was used to extract total RNA from different tissues and feces samples according to the manufacturer's instructions. A PrimeScript RT reagent Kit with gDNA Eraser (TaKaRa, Japan) was applied to conduct reverse transcription. A QuantiTect Probe PCR Kit (Qiagen, USA) was used for perform the quantitative real-time PCR in a 25 μl reaction volume. The thermal cycling profile below was made by an ABI 7500 Fast Real-Time PCR System (Applied Biosystems): initial denaturation at 95°C for 15 min, then 40 cycles of 95°C for 10 s and 60°C for 1 min.

### Ethics Statement

The Experimental Animal Ethics Committee (North China University of Science and Technology) approved this animal infection experiment, and the animals were maintained in accordance with the guidelines issued by North China University of Science and Technology for the care and use of laboratory animals.

## Results

### MNV Detection in Commercial Laboratory Mice

The presence of MNV was checked by testing fecal specimens from 170 C57BL/6J mice. Among 17 laboratory mice pooled samples, RT-PCR revealed 15 samples positive for MNV by RT-PCR (Supplementary Table 1 and Supplementary Figure 1), and all of the mice pooled samples from Shandong and Liaoning Province were detected positive for MNV.

### Isolation and Identification of the MNV

**Figure 1:**
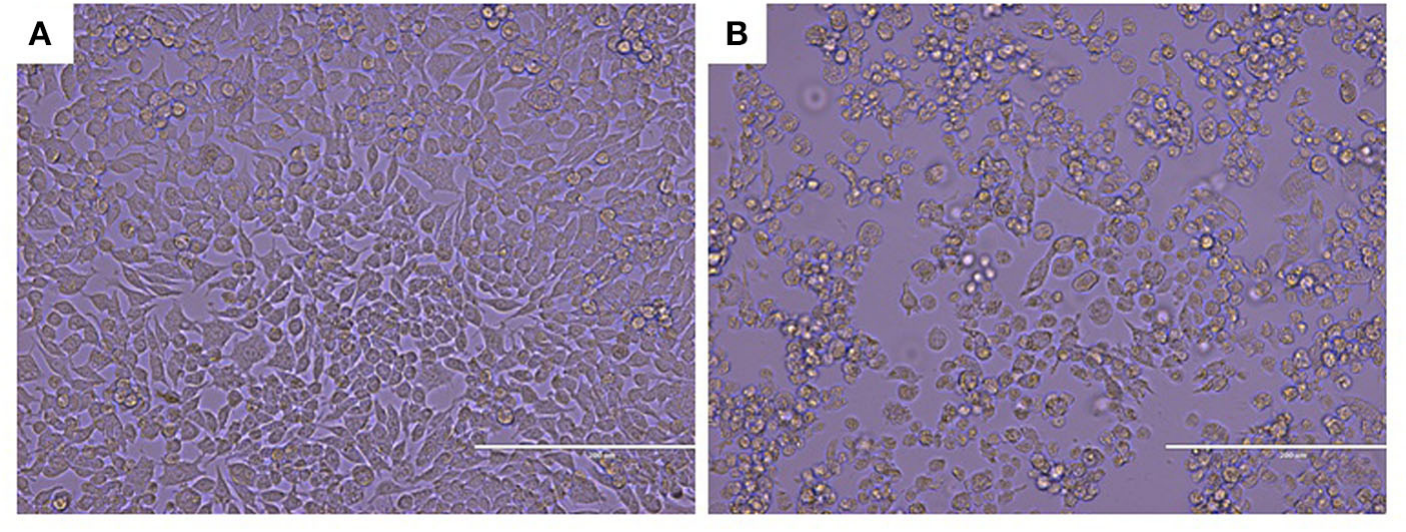
CPE in MNV infected RAW264.7 cell (Original magnification × 200). **(A)** Non-infected RAW264.7 cell monolayer; **(B)** Infected Vero cells rounded up and focal detachment 48 h post-infection.

**Figure 2:**
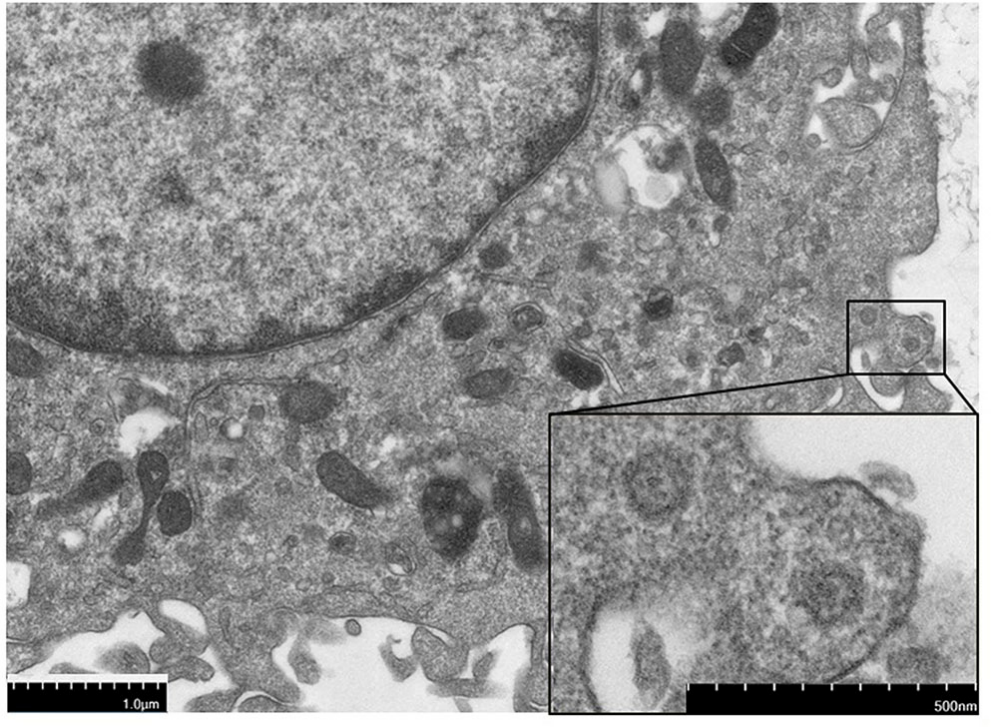
The electron micrographs of virus particles. The section of the infected cell 24 h post-infection, showing dense particles and virions.

Since the virus could replicate in RAW264.7 cells, infected cells rounded up and drifted out of the flask surface and focal CPE appeared 48 h after infection (Figure 1). The newly isolated virus was named MNV HBTS-1806. 24 h after infection an ultrathin section of infected RAW cells was obtained. EM identified dense particles with the diameter of 50–70 nm within cytoplasmic vesicles (Figure 2). Human norovirus appeared similar morphology as ~50 nm in diameter (29). All the passages of the cell cultures were tested positive for MNV by RT-PCR.

### Persistent Infection With the Isolated Virus in Wild-Type Mice

All mice infected by the MNV HBTS-1806 strain *via* oral gavage inoculation at a dose of 5 × 10^5^ TCLD_50_/mouse exhibited no significant clinical signs until 12 weeks post-infection. There are no significant difference in food intake and body weight between two groups. To monitor viral RNA in some organs of all infected mice between 2 and 12 weeks after infection, the viral RNA was extracted and analyzed from stomach, appendix, liver, spleen, and feces at different time points (Table 2). Six of ten appendices were still positive12 weeks after infection. Tissues from mice sacrificed at 2, 4, and 12 weeks were examined histologically after infection. There is no apparent microscopic change in the liver, spleen and appendix (Supplementary Figure 2).

**Table 2:** MNV RNA detection in tissues of 6 week-old C57BL/6J mice tissues sacrificed at various time-points.

**Weeks**	**Tissues samples**			
**post-infection**	**Stomach**	**Appendix**	**Liver**	**Spleen**
2	80% (10/10)	100% (10/10)	50% (5/10)	70% (7/10)
4	40% (4/10)	100% (10/10)	10% (1/10)	10% (1/10)
6	20% (2/10)	100% (10/10)	0% (0/10)	0% (0/10)
8	10% (1/10)	80% (8/10)	0% (0/10)	0% (0/10)
10	10% (0/10)	80% (8/10)	0% (0/10)	0% (0/10)
12	10% (0/10)	60% (6/10)	0% (0/10)	0% (0/10)

### Genomic Sequencing and Phylogenetic Analysis

**Figure 3:**
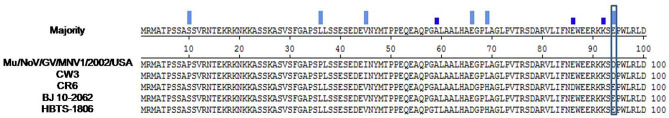
Align sequencs of 1–100 domain of MNV NS1/2 protein construts. The presence of a glutamate at position 94 of the NS1/2 protein could played an essential role in persistent replication and shedding of MNV.

**Figure 4:**
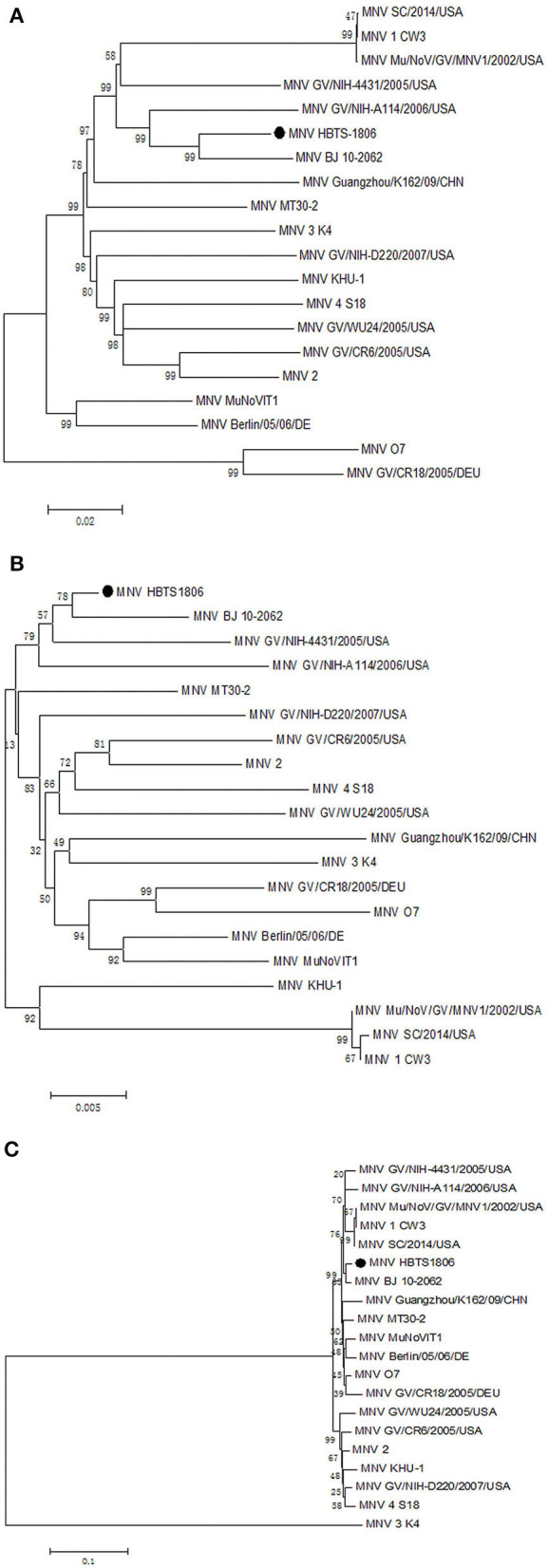
Phylogenetic relationships of the isolated MNV with other MNVs. Nucleotide sequence comparisons of the whole genome **(A)**, ORF1 **(B)**, and ORF2 **(C)**.

The entire genome of MNV HBTS-1806 strain was sequenced and compared with the full-length sequences of reference MNVs (Table 3). The HBTS-1806 contained 7383 nucleotides and had four ORFs, conforming to the genomic characteristics of MNV1. The genetic distances between the query strain and the reference MNV strains were drawn according to nucleotide position. The overall sequence similarity among these MNVs was 88.9–95.4%. Highest homology to isolates was the MNV BJ-2011-1 strain, which was isolated in Beijing of China. The glutamate at position 94 in the NS1/2 protein was critical for persistent replication and shedding (Figure 3). Phylogenetic analysis based on ORF1 sequences placed HBTS-1806 away from MNV1 (Mu/NoV/GV/MNV1/2002/USA) (Figure 4B), while the tree based on the whole genome and ORF2 showed close evolutional relationship between HBTS-1806 and MNV1 (Mu/NoV/GV/MNV1/2002/USA) (Figures 4A,C).

**Table 3:** MNV strains investigated in this study.

**Strain**	**GenBank no**.	**Country**
Mu/NoV/GV/MNV1/2002/USA	AY228235	USA
GV/NIH-4431/2005/USA	JF320651	USA
GV/CR6/2005/USA	EU004676	USA
GV/CR18/2005/DEU	EU004683	USA
GV/WU24/2005/USA	EU004669	USA
CW3	EF014462	USA
GV/NIH-A114/2006/USA	JF320652	USA
MNV2	DQ223041	USA
GV/NIH-D220/2007/USA	JF320653	USA
Berlin/05/06/DE	EF531290	GER
MNV 3 K4	FJ446720	ROK
MNV 4 S18	FJ446719	ROK
Guangzhou/K162/09/CHN	HQ317203	CHN
MT30-2	AB601769	JPN
KHU-1	JX048594	CHN
O7	KF113526	GBR
BJ 10-2062	KM458057	CHN
SC/2014/USA	KM102450	USA
MuNoVIT1	KR349276	ITA

## Discussion

Murine noroviruses consist of a group of newly-recognized pathogens that are able to infect laboratory mice. The first reported MNV-1 generates a transient infection with a short duration of fecal shedding after infecting immunocompetent mice in laboratory (30). It is very likely that MNV infection can be widespread across the world (11). Besides, once infected the host, MNV is highly persistent and hard to be removed completely without an integrated eradication system (31). MNV isolates such as MNV-2, MNV-3, and MNV-4,have been extractedfrom laboratory mice in academic research institutes around the world (8). There are dramatic differences between these MNV strains and MNV-1 in pathogenicity because MNV-1 persistently infect tissues in experimentally inoculated immunocompetent mice (32). Therefore, it is necessary to screen laboratory animals for MNV frequently to prevent animal diseases and interfere in experiments (8, 33, 34). This stduy describes a novel strain of MNV isolated from feces samples of commercial laboratory mice. The virus was successfully propagates in RAW264.7 cell culture. And the size and morphological characteristics of this virus were similar to norovirus described previously by other investigators (35, 36).

In general, different MNV isolates show different pathogenicity. A recent research reported that an MNV strain (CR6) can cause intestinal pathology under a specific Crohn's disease genetic background, while another strain (MNV-1) cannot (37). In this work, MNV HBTS-1806 strain infection of immunocompetent C57BL/6J mice did not cause gross pathology. Moreover, no lesion was found in small intestine, liver or spleen of animals in this study. Although no significant pathological changes in immunocompetent mice were shown, it should be noted that infectious viral titers in tissues were continuously detected 2–12 weeks after infection. And even virus loads in the intestinal would last 12 weeks. Our data demonstrated that intestinal pathology cannot be predicted by MNV titers in wild-type mice, which could be potentially explained by that to the specificity of virus strains in either cell tropism relating to virulence or interactions with host cells (12). Since the biological significance of these observation is not completely understood yet, the situation that the prolonged viral load may be a major risk factor for large-scale MNV infection in the animal breeding and housing environment shall be recognized. Because the MNV was described as an acute infection with apparent mortality and morbidity in Stat1^−/−^ mice, persistent subclinical infection in Rag^−/−^ mice, the pathogenicity of isolates in immunodeficient mice requires further study (11).

How persistent MNV infection is maintained is unclear. During persistent infection, the mutations of MNV surface antigens were predicted to make viral progeny avoid the immune system (38). In addition, genetic elements in the NS1/2 protein causing persistence of the virus in the mouse determine the viral persistence in the intestinal tract. Residue 94, a critical factor determining persistence, was separated in a reverse turn after an α-helix in the folded domain (39). The presence of glutamate at position 94 of the NS1/2 protein from HBTS-1806 plays an essential role in persistent replication and shedding.

This study reported the complete genomic sequence of HBTS-1806 and analyzed the phylogenetic associations among MNV strains on basis of the genomic and individual gene levels, so as to characterize the novel isolated virus. Sequence homology and phylogenetic analysis of the MNV showed that the HBTS-1806 strain has high homology with MNV BJ-2011-1. The alignments of the whole genome, ORF1, and ORF2 nucleotide sequences of the HBTS-1806 strain and MNV1 (Mu/NoV/GV/MNV1/2002/USA) strain revealed considerable distinctions among the trees. The recombinantion of human noroviruses have been recognized as the cause of global norovirus outbreak (40–42). Nevertheless, the molecular mechanisms promoting norovirus recombination have not been determined experimentally (43). The report suggested that the recombination may occurred at several points of breakout within ORF2 in several MNV genomes, despite of the predominant break point detected in recombinant human norovirus genomes is at the ORF1-ORF2 junction (44). However, contribution of recombination to the evolution of the MNV strains is not known, and the effects of the recombination on MNV biology are to be elucidated in the future.

## Data Availability Statement

The datasets generated in this study can be found in online repositories. The names of the repository/repositories and Accession Number(s) can be found below: https://www.ncbi.nlm.nih.gov/, MT358379.

## Ethics Statement

The animal study was reviewed and approved by the Experimental Animal Ethics Committee (North China University of Science and Technology).

## Author Contributions

LS and SJ performed experiments, analyzed data, and wrote the manuscript. ZN, JB, YY, and CF carried out the experiments. HB and JH participated in the design. ZY and JB participated in the design and analysis of data of sequence. JB and ZN participated in manuscript drafting. All authors contributed to the article and approved the submitted version.

## Conflict of Interest

The authors declare that the research was conducted in the absence of any commercial or financial relationships that could be construed as a potential conflict of interest.
