# Supplementary figures and images for: Isolation and Identification of a Murine Norovirus Persistent Infection Strain in China
Source: Front Vet Sci. 2020 Dec 1;7:571730. doi: 10.3389/fvets.2020.571730 (PMC7736604; doi:10.3389/fvets.2020.571730)

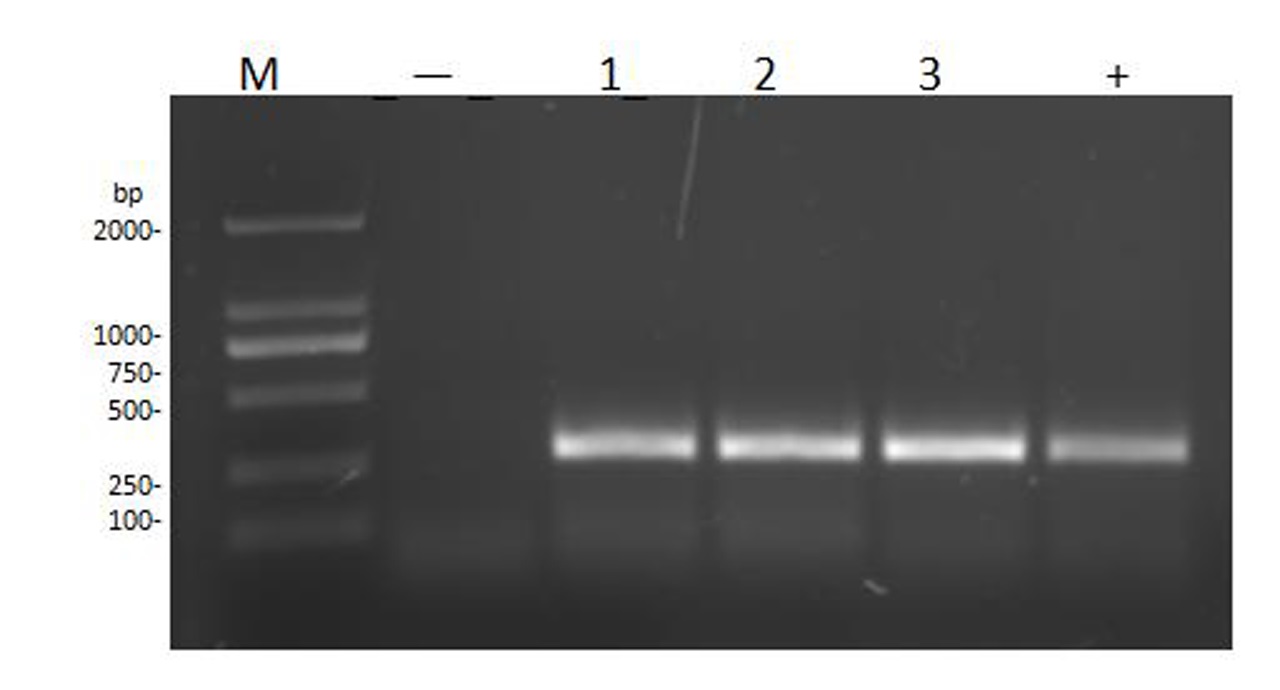

Supplement: Supplementary file 1 [file Image_1.jpeg]

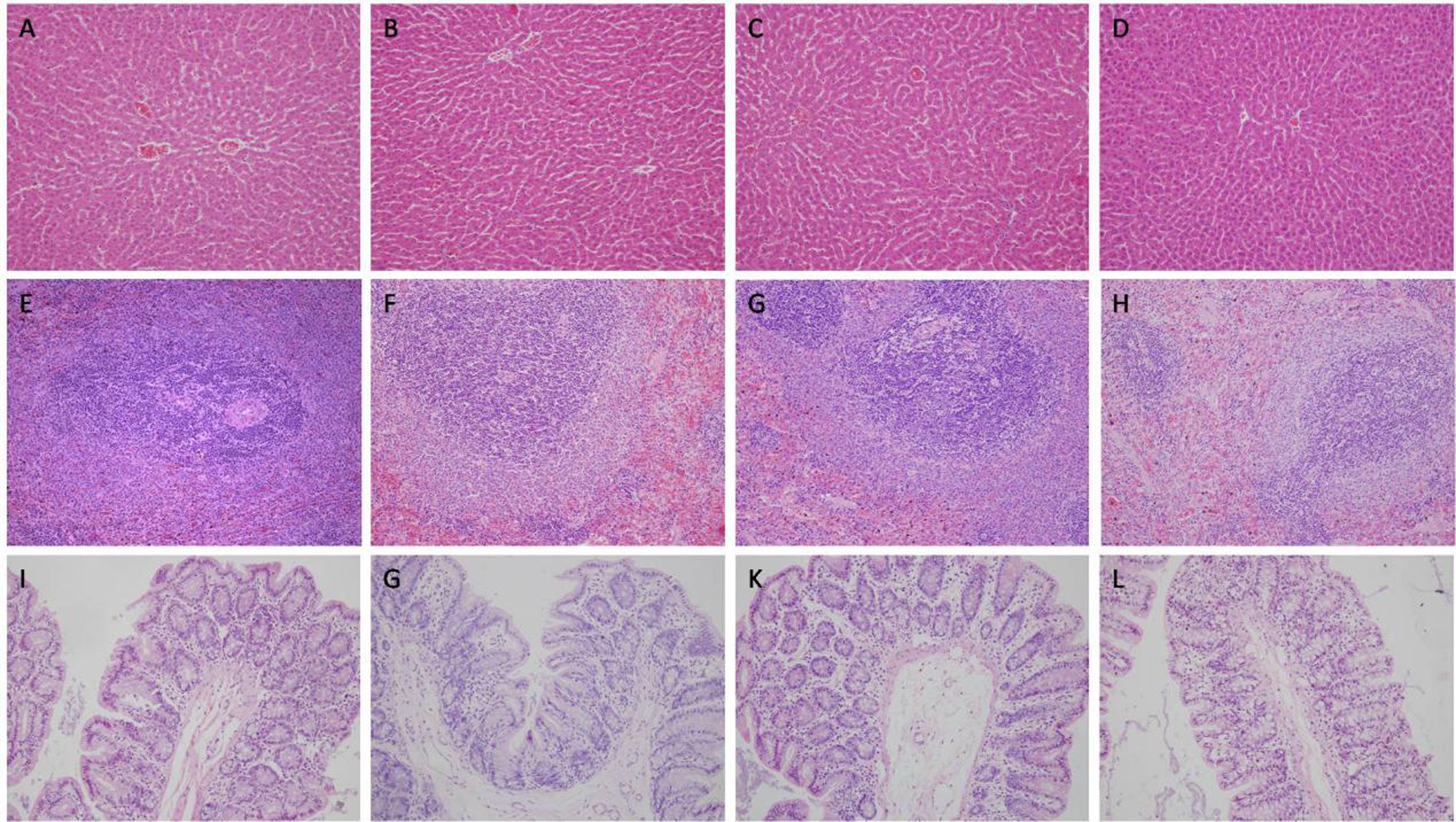

Supplement: Supplementary file 2 [file Image_2.jpeg]
