# Supplementary material for: Isolation and Identification of a Murine Norovirus Persistent Infection Strain in China
Source: Front Vet Sci. 2020 Dec 1;7:571730. doi: 10.3389/fvets.2020.571730 (PMC7736604; doi:10.3389/fvets.2020.571730)
Supplement: Supplementary file 3 [file Table_1.docx]

**S 1** MNV detected in the fecal specimens of C57BL/6J mice by RT-PCR

| Area | date | Sample | PCR | Positive (%) |
| --- | --- | --- | --- | --- |
| Shandong Province | Mar 3 | 10 | + | 100% |
|  | Apr 15 | 10 | + |  |
|  | May 20 | 10 | + |  |
|  | Jun 5 | 10 | + |  |
|  | Jul 10 | 10 | + |  |
|  | Sep 30 | 10 | + |  |
| Liaoning Province | Mar 20 | 10 | + | 100% |
|  | May 5 | 10 | + |  |
|  | May 30 | 10 | + |  |
|  | Apr 8 | 10 | + |  |
|  | Jun 2 | 10 | + |  |
|  | Sep 12 | 10 | + |  |
| Beijing | Apr 1 | 10 | + | 40% |
|  | Jun20 | 10 | - |  |
|  | Jul 21 | 10 | + |  |
|  | Aug 4 | 10 | - |  |
|  | Sep 3 | 10 | - |  |
